# Nano-immunotherapy for each stage of cancer cellular immunity: which, why, and what?

**DOI:** 10.7150/thno.59953

**Published:** 2021-06-01

**Authors:** Shiyi Zuo, Jiaxuan Song, Jingxuan Zhang, Zhonggui He, Bingjun Sun, Jin Sun

**Affiliations:** Department of Pharmaceutics, Wuya College of Innovation, Shenyang Pharmaceutical University, Shenyang 110016, China.

**Keywords:** Cancer immunotherapy, nanotechnology, cellular immunity, immunity cycle, combination therapy.

## Abstract

Immunotherapy provides a new avenue for combating cancer. Current research in anticancer immunotherapy is primary based on T cell-mediated cellular immunity, which can be divided into seven steps and is named the cancer-immunity cycle. Unfortunately, clinical applications of cancer immunotherapies are restricted by inefficient drug delivery, low response rates, and unmanageable adverse reactions. In response to these challenges, the combination of nanotechnology and immunotherapy (nano-immunotherapy) has been extensively studied in recent years. Rational design of advanced nano-immunotherapies requires in-depth consideration of “which” immune step is targeted, “why” it needs to be further enhanced, and “what” nanotechnology can do for immunotherapy. However, the applications and effects of nanotechnology in the cancer-immunity cycle have not been well reviewed. Herein, we summarize the current developments in nano-immunotherapy for each stage of cancer cellular immunity, with special attention on the which, why and what. Furthermore, we summarize the advantages of nanotechnology for combination immunotherapy in two categories: enhanced efficacy and reduced toxicity. Finally, we discuss the challenges of nano-immunotherapy in detail and provide a perspective.

## Introduction

Cancer is one of the most severe diseases threatening human health. Chemotherapy, surgery, and radiotherapy are the three standard clinical treatments for cancer. These conventional treatments can extend patient survival, but they are constantly challenged by intractable problems, including severe adverse reactions, inevitable tumor recurrence, and resistance 1. In recent years, cancer immunotherapy has developed as the fourth treatment modality. Cancer immunotherapy evokes or boosts the inherent host immune system and then enhances antitumor immune responses, providing a new avenue to combat cancer 2.

**Figure 1:**
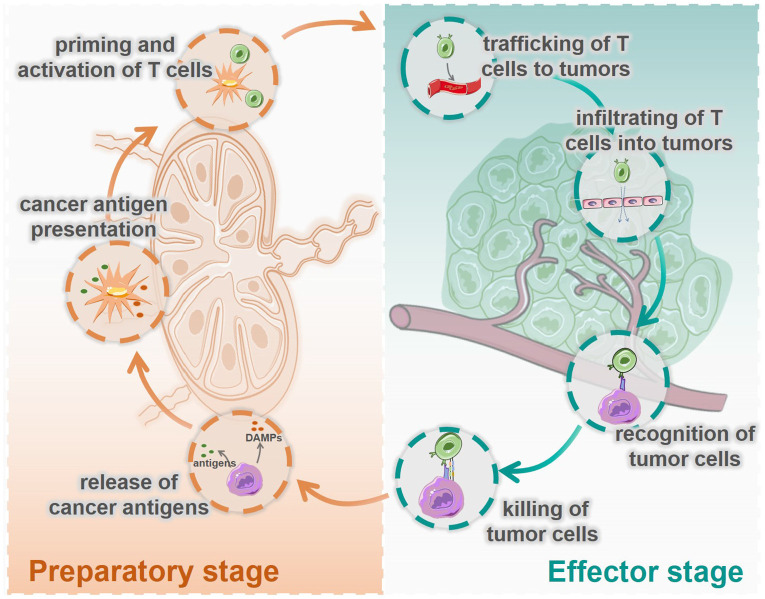
Seven immune actions in the cycle of cancer cellular immunity are divided into two stages, the preparatory stage and the effector stage. The preparatory stage (the left side) includes the release of cancer antigens (step 1), cancer antigen presentation (step 2), priming and activation of T cells (step 3), which mainly take place in the lymph nodes. The effector stage (the right side) includes trafficking of T cells into tumors (step 4), infiltration of T cells into tumors (step 5), recognition of cancer cells (step 6), and killing of cancer cells (step 7), which mainly happen in TME.

At present, most cancer immunotherapies are based on T cell-mediated cellular immunity 3, 4, which has been defined as the famous cancer-immunity cycle by Chen and Mellman 5. As shown in Figure 1, this cycle includes seven steps: 1. release of cancer cell antigens; 2. cancer antigen presentation; 3. priming and activation of T cell; 4. trafficking of T cells to tumors; 5. infiltration of T cells into tumors; 6. recognition of cancer cells by T cells; and 7. killing of cancer cells. These seven steps can be divided into two stages: the preparatory stage (T cells responding) consisting of steps1 to 3, which mainly take place in the lymph nodes (LNs), and the effector stage (T cells killing) consisting of steps 4 to 7, which mainly take place in the tumor microenvironment (TME). In most tumors, the cancer-immunity cycle is blocked at one or more of these steps, resulting in restrained anticancer immune responses and tumor immune escape.

Despite huge breakthroughs, immunotherapy is still limited by unsatisfactory response rates, efficacy and safety 6, 7. First of all, many immunotherapy agents suffer from low solubility, poor stability, and short half-lives 8. Secondly, some immunotherapies can cause severe or even fatal allergy- and inflammation-related reactions 9. These reactions happen when the immune systems not only fight cancer but also attack healthy cells and tissues in the body 10. It is also challenging to deliver immune cells or agents into tumors through an immunosuppressive tumor microenvironment (iTME) 11. Moreover, the low immunogenicity of tumor cells and the accumulation of immunosuppressive cells and cytokines in the iTME together limit the effects of immunotherapies 12. Therefore, the rational design of advanced antitumor immunotherapies is still a great challenge.

Nano-drug delivery systems have been widely used in the field of anticancer therapy 13. Nanoparticles (NPs) can increase the stability of drugs and protect them from being metabolized during blood circulation, thus enabling reduction of the administered dose and avoidence of high dose-related toxicities. Moreover, NPs can increase the accumulation of therapeutic agents in tumor tissue and LNs, leading to enhanced therapeutic effects and reduced side effects 14. It is well known that tumors and LNs are the two main targets of immunotherapy 15, 16. NPs can passively transport into tumor tissue through immature tumor vasculature and accumulate due to damaged lymphatic drainage, a phenomenon known as the enhanced permeability and retention (EPR) effect 17. Also, NPs can actively target tumor cells after surface ligand modification 18. Similarly, NPs can accumulate in LNs and deliver cancer vaccines to antigen-presenting cells (APCs) to activate an immune response 19-23. What is more, NPs have distinct advantages for combination drug delivery, which can synergize multiple immunotherapy mechanisms to enhance the overall immune response 24-28. Based on the above advantages, nano-immunotherapy has become a hot topic in recent years.

**Figure 2:**
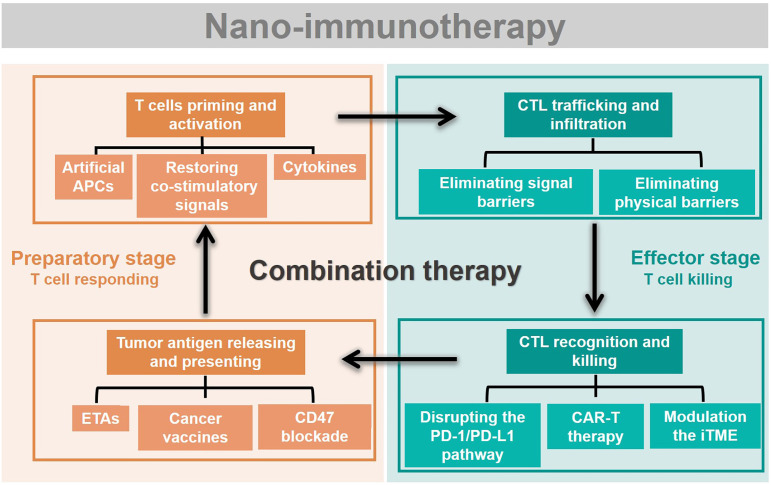
Immunotherapies combing with nanotechnology. The seven steps of the cancer-immunity cycle are simplified into four parts. To present which immune step is associated, the current developments of nano-immunotherapy are divided into these four parts. Nano-immunotherapies acting on preparatory stage include tumor antigen releasing and presenting (by ETAs, cancer vaccines, CD47 blockade therapy), and T cells priming and activation (by artificial APCs, restoring co-stimulatory signals, cytokines therapy). The functions of nano-immunotherapies acting on the effector stage are CTL trafficking and infiltration (through eliminating physical barriers and signal barriers), and CTL recognition and killing (through disrupting the PD-1/PD-L1 pathway, CAR-T therapy and modulation the iTME). Besides, combination nano-immunotherapy consists of immunotherapies with different effects, making up for the deficiency of monotherapy in the immunity cycle.

Rational design of advanced nano-immunotherapies requires in-depth consideration of “which” immune step is targeted, “why” it needs to be further enhanced, and “what” nanotechnology can do for immunotherapy. However, the applications and effects of nanotechnology in each stage of cancer immunity have not been systematically summarized. Herein, we streamline the seven steps of the cancer-immunity cycle into four parts according to their correlations (Figure 2): tumor antigen (TA) release and presentation (steps 1 and 2); T-cell priming and activation (step 3); cytotoxic T lymphocyte (CTL) trafficking and infiltration into the tumor site (steps 4 and 5); and CTL recognition and killing of tumor cells (steps 6 and 7). For each of these four parts, we summarize the current developments in nano-immunotherapy. In this way, which immune step is associated with a specific nano-immunotherapy is clear. In addition, the shortcomings of each immunotherapy are summarized based on preclinical and clinical research reports to explain why it needs to be further enhanced. The benefits and improvements that nanotechnology can provide to each immunotherapy are then presented. Moreover, recent preclinical and clinical studies have explored combinations of immunotherapies that target different pathways, which could be improved by nanotechnology 29. Thus, we summarize the advantages of nanotechnology for combination immunotherapy. Finally, we discuss the challenges of nano-immunotherapy and provide our perspective on the future of the field.

## Antigen release and presentation

Cellular immunity starts with the release and exposure of TAs, which are then captured by APCs such as dendritic cells (DCs) 30. After migrating to draining LNs, DCs mature and subsequently present the antigens to naive T cells via major histocompatibility complex (MHC) I and II molecules 31. Release and presentation of TAs are the preconditions of cellular immune response. However, many tumors have poor immunogenicity due to down-regulation of antigen expression, antigen loss, and antigen modulation 32, 33. Also, antigen presentation to T cells by dysfunctional DCs induces antigen-specific immunotolerance. These traps impede initiation of T cell-mediated immunity. Plenty of immunotherapies have been developed to improve the release and presentation of TAs, including induction of endogenous tumor antigens (ETAs) 34, 35, cancer vaccines, 36 and blockade of the CD47 immune checkpoint 37.

### Inducing endogenous tumor antigens

ETAs are able to arouse specific immune responses for personalized immunotherapy 38. Treatments such as photodynamic therapy (PDT), photothermal therapy (PTT), certain chemotherapies, and radiotherapy have been reported to induce endoplasmic reticulum (ER) stress and immunogenic or necrotic tumor cell death, leading to release of ETAs from tumor cell residues 39. However, the rapid immune clearance and inefficient delivery of these autologous antigens restrict stimulation of an antitumor immune response 40. Rational design of nanocarriers can protect antigens from clearance and target their delivery to LNs. For example, Qian et al. developed mesoporous silica nanoparticles (MSNs) incorporating NP debris combined *in situ* with ETAs from tumor cells killed by PTT. Then, the nano-debris carried the antigens out of the necrotic tissue and selectively entered the immune organs for immunotherapy 41. Similarly, Wang et al. found that ~10 nm iron oxide (Fe_3_O_4_) can transport ETAs from tumor to LNs because of their notable protein capture efficiency and LN-targeting ability. To ensure efficient combination of Fe_3_O_4_ with ETAs, a core-shell nanostructure (denoted as Ce6/Fe_3_O_4_) was developed to protect the Fe_3_O_4_ core from interaction with undesired proteins. Upon laser irradiation, reactive oxygen species (ROS) generated by the outer chlorin e6 (Ce6) photosensitizer triggered release of ETAs from cancer cells. Fe_3_O_4_ then captured the released ETAs and transported them to LNs via lymphatic drainage 34.

Induction of ER stress often leads to immunogenic cell death (ICD) 42, 43. ICD can enhance adjuvanticity and antigenicity from dying cancer cells by releasing damage-associated molecular patterns (DAMPs), including calreticulin (CRT), high mobility group box 1 (HMGB1), and adenosine triphosphate (ATP) 44. These DAMPs facilitate recruitment and maturation of DCs, thus improving antigen presentation. PDT is a common strategy to induce ICD of tumor cells. However, ICD induced by conventional PDT is reported to be less immunogenic than necrosis due to the slow release of DAMPs. Moreover, low PDT efficacy limits the release of ETAs. Zhang's group designed a tumor cell plasma membrane (PM)-targeted chimeric peptide, PpIX-C6-PEG8-KKKKKKSKTKC-OMe (PCPK), containing the photosensitizer protoporphyrin IX (PpIX) to achieve specific PM damage for enhanced photodynamic immunotherapy. In tumor cells, PCPK tightly anchored to the inner PM after protein farnesyltransferase (PFTase)-mediated enzymatic conversion. Under laser irradiation, PCPK generated cytotoxic ROS, which destroyed the structure of the PM. In addition, the selective rupture of the PM induced rapid release of ETAs and DAMPs, leading to significantly enhanced antitumor immune response compared to conventional PDT 45.

In summary, nanotechnology-based treatments inducing antigen release have been shown to enhance antigen production, retention and LN targeting.

### Cancer vaccines

Cancer vaccines composed of TAs and adjuvants are widely used to induce an immune response 46, 47. The major challenges of cancer vaccines include drainage of antigens to LNs, internalization of antigen by DCs, and cross-presentation of antigens by DCs for T-cell activation 36, 48, 49. It has been found that NPs of a suitable size can accumulate in tumor-draining lymph nodes (TDLNs) following tumor accumulation by the EPR effect 19, 50. Also, a negative surface charge can facilitate lymphatic uptake and retention of NPs through electrostatic interactions 51. For example, MSNs have been extensively studied as antigen carriers because of their ultra-high surface area, easy surface modification, and adjustable particle size 52. In addition, the surface of MSNs is negatively charged and contains hydrophilic silanol groups (Si-OH), making MSNs a potential LN-targeted carrier 53, 54. Hong et al. constructed three types of MSNs with similar particle sizes (~80 nm) but different pore sizes and evaluated their ability to deliver a model antigen (ovalbumin, OVA) to TDLNs. The authors found that all three OVA@MSNs accumulated in TDLNs and were then internalized by LN-resident DCs to a similar degree, probably because their similar particle sizes. Interestingly, the MSNs with the largest pore size (MSNs-L) improved the cross-presentation of OVA. In addition, MSNs-L were degraded faster in TDLNs than the other MSNs, which may have facilitated OVA release and exposure. Rapid degradation of MSNs-L also helped to improve their safety *in vivo*. As a result, MSNs-L significantly enhanced the delivery efficiency of OVA and induced more robust immune responses than the MSNs with smaller pores 55.

Antigen presentation to T cells by dysfunctional DCs induces antigen-specific immunotolerance. Adjuvants, including particulate adjuvants (such as alum and emulsions) and pattern recognition receptors (PRRs) agonists, are applied to cancer vaccines to improve maturation of DCs 56-59. Among them, PRRs agonists, the new-generation adjuvants, have been used to activate myeloid cells (macrophages and DCs) and induce antitumor immune responses 60-62. However, application of PRRs agonists is restricted by serious adverse effects, which are mainly caused by their unfavorable pharmacokinetic profiles and biodistribution 63, 64. Ni et al. reported a bi-adjuvant neoantigen nanovaccine (banNV) that co-delivered a peptide neoantigen (Adpgk) with the Toll-like receptor (TLR) 7/8 agonist R848 and the TLR9 agonist CpG. Through efficient co-delivery of the neoantigen and the dual synergistic adjuvants, the immunogenicity of the neoantigen was potentiated, and acute systemic toxicity was reduced 65.

To improve the targeting of cancer vaccines to APCs, NPs modified with specific receptors for APCs have been applied as carriers of cancer vaccine. For example, Affandi et al. described a nanovaccine targeting CD169/SIGLEC1^+^ DCs to drive antitumor T-cell responses. The authors used the natural ligands of CD169, gangliosides, as targeting ligands and constructed liposomal vaccine carriers to deliver TAs. Through targeted co-delivery of TA and TLR ligand to CD169^+^ monocyte-derived DCs and AXL^+^CD169^+^ DCs, the ganglioside-liposomes induce robust cross-presentation and activation of TA-specific CD8^+^ T cells 66. Rajpu et al. engineered polymeric nanovaccines using inulin acetate (InAc) as the polymer material. DCs recognized InAc-NPs through TLR4-InAc interactions, which led to efficient vaccine uptake, antigen presentation, and TLR4-based signaling, improving DC activation and maturation 67. Yang et al. constructed a nanovaccine (NP-R@M-M) by coating R837-loaded poly(lactic-*co*-glycolic acid) (PLGA) NPs with mannose-modified B16-OVA membranes. Owing to their strong interaction with DCs (mannose receptor positive), NP-R@M-M showed enhanced uptake by DCs and stimulation of DC maturation compared with untargeted NPs. Although NP-R@M-M exhibited strong antitumor efficacy in a B16-OVA melanoma tumor model, it did not show any antitumor efficacy in a 4T1 mouse breast tumor model, evidencing the specificity of the antitumor immunity induced by NP-R@M-M. Therefore, NP-R@M-M demonstrated a strong stimulation effect for triggering antitumor immune responses with good specificity and safety 21.

mRNA vaccines represent a promising alternative to conventional vaccines 68. Instead of directly delivering pathogen proteins, mRNA vaccines trigger endogenous production of immunogenic proteins, thus inducing a strong MHC-I-mediated CD8^+^ T-cell response and reducing adverse reactions 69. However, the delivery of mRNA vaccines to APCs is challenged by the following issues: (a) degradation by omnipresent endonucleases, (b) difficulty in accessing APCs, and (c) endosomal entrapment and degradation. Recently, ionizable lipid nanoparticles (LNPs) have emerged as promising platforms for efficient mRNA delivery 2, 70-72. Oberli et al. developed LNPs encapsulating mRNA coding OVA. As lipids are the basic units of biomembranes, the LNPs had a good affinity with PM and facilitated cellular uptake of mRNA. Within acidic intracellular environments (such as lysosomes), the ionizable lipid core was positively charged, thereby integrating with the negatively charged mRNA and then protecting it from degradation. In addition, the positively charged LNPs facilitated escape of the mRNA from lysosomes via the proton sponge effect. Treatment of a B16-F10 melanoma tumor model with the LNPs resulted in tumor shrinkage and extended overall survival 73.

In brief, nanotechnology can improve the efficacy of cancer vaccines by protecting the antigen/adjuvant from clearance in the biological environment, promoting delivery of the antigen/adjuvant to LNs and APCs, and minimizing systemic toxicity.

### CD47 blockade

CD47 transmits an inhibitory “don't eat me” signal upon ligation with its receptor signal regulatory protein alpha (SIRPα), which is mainly expressed on phagocytic cells, including macrophages and DCs 74. Recent studies have demonstrated that CD47 blockade not only increases phagocytosis of cancer cells by macrophages but also promotes cross-presentation of TAs 75. Blockade of the immune checkpoint with antibodies has been extensively evaluated in the clinical setting. Nanobodies have been developed to improve the therapeutic efficacy of conventional antibodies, with distinct advantages such as high stability, high affinity and specificity, and deep tissue penetration 10, 76. Chowdhury et al. engineered a non-pathogenic *Escherichia coli* strain that specifically lysed within the TME and released an encoded nanobody antagonist of CD47 (CD47nb). Compared with the commonly used anti-mouse CD47 monoclonal antibody, the produced CD47nb had a higher binding affinity to CD47 on the surface of A20 lymphoma cells. Tumors treated with CD47nb showed increased proliferation of both FOXP3^-^CD4^+^ and CD8^+^ T cells. These results suggested that compared to conventional systemic monoclonal antibody therapy, localized release of the CD47nb increased the activation of tumor-infiltrating T cells and simultaneously prevented systemic toxicity 75.

## T cells priming and activation

**Figure 3:**
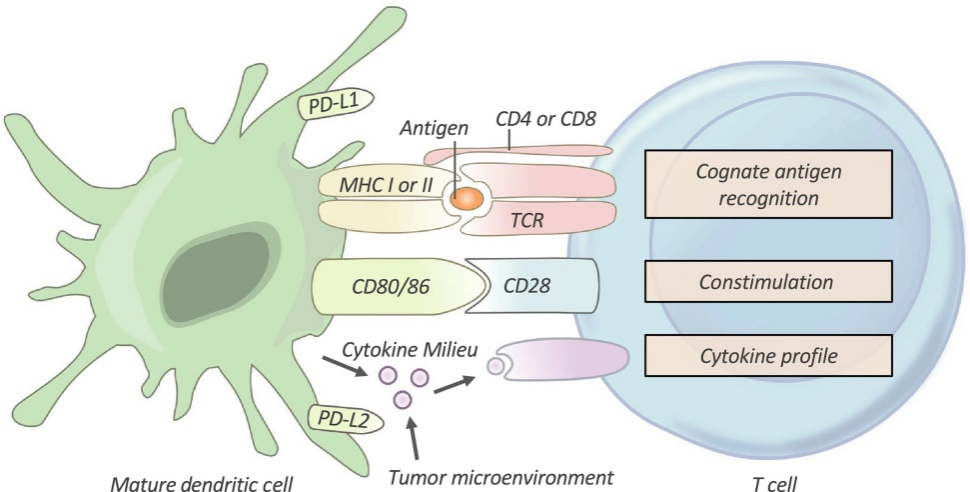
T-cell Activation. T cells require three signals from an antigen‐presenting cell (APC) in order to be effectively activated and primed. First, T cells must recognize their cognate antigen in the context of the correct MHC. Second, T cells require appropriate co-stimulation by an APC. Finally, the T cell receives instructive cytokines from immune cells and the tumor microenvironment which dictate its phenotypic differentiation. TCR, T-cell receptor; PD-L1/PD-L2, programmed death-ligands 1 and 2. Adapted with permission from 4, Copyright 2019 Clinical & Translational Immunology.

After being taken up by DCs, TAs are processed to peptide antigen-loaded major histocompatibility complex (pMHC). Immature T cells are then primed and activated to become effector T cells 5, 13. As shown in Figure 3, priming and activation of T cells requires three signals from APCs: antigen recognition between T cells and APCs (signal 1), a co‐stimulatory signal (signal 2), and a cytokine signal (signal 3). However, in most cases, the T cells of cancer patients are activated without the combination of these signals. Therefore, the T cells usually become nonresponsive to further stimulation and stay in a state of anergy. Artificial APCs (aAPCs), restoration of co-stimulatory signals, and cytokine therapies, have been utilized to facilitate activation of T cells 4, 60, 77.

### Artificial APCs

The inefficient presentation of TAs by immature APCs leads to deficiency of signal 1 and further restricts activation of CTLs 78. aAPCs are microparticle- or NP-based biomimetic systems that contain pMHC complexes and positive co-stimulatory molecules on their surface. aAPCs can directly stimulate antigen-specific CTLs, bypassing the need for endogenous APCs 79, 80. Hickey et al. demonstrated that aAPCs larger than 300 nm were more effective at activating CD8^+^ T cells than smaller aAPCs, presumably due to their ability to initiate clustering of pMHC-T cell receptor (TCR) complexes and costimulatory interactions 81. Green's group showed that non-spherical aAPCs induced more robust antigen-specific T-cell responses than spherical aAPCs 80. In addition, Majedi et al. combined mechanical forces with aAPCs and confirmed that exogenous mechanical forces increased the antigenic signal strength to T cells 82. Magnetic aAPCs with an externally applied magnetic field can further improve T-cell activation. Zhang et al. developed biomimetic magnetosomes as versatile aAPCs. The magnetic nanoclusters were first coated with azide-engineered leucocyte membranes, then loaded with pMHC-I and the co-stimulatory ligand anti-CD28 for T-cell stimulation. These nano aAPCs exhibited a high performance for antigen-specific CTL expansion and stimulation and showed a good affinity for CTLs. Additionally, through magnetic resonance imaging and magnetic control, these biomimetic aAPCs efficiently guided reinfused CTLs to tumor tissues 83.

In summary, nanotechnology improved the LN targeting and safety of aAPCs. However, nanoscale aAPCs have a decreased surface area for contact with T cells compared with microscale aAPCs, which would affect their T-cell activation efficacy.

### Restoring co-stimulatory signals

Interaction between the co-stimulatory molecules CD28 (on T cells) and B7 (on APCs) provides a co-stimulatory signal (signal 2) that is required for T-cell activation. Cytotoxic T lymphocyte antigen 4 (CTLA-4) small interfering RNA (siCTLA-4) utilizes endogenous RNA interference mechanisms to silence CTLA-4 expression, thereby promoting T-cell activation. However, the efficiency of siRNA is restricted by its large molecular weight, rapid degradation by plasmatic nucleases, and extracellular barriers. LNPs are capable of dealing with these problems. With their capacity to condense and protect siRNA and their good cellular internalization efficiency, LNPs have been developed as the gold standard gene vector for delivery of siRNA. Li et al. prepared cationic lipid-assisted poly(ethylene glycol)-polylactide (PEG-PLA) NPs to encapsulate siCTLA-4 (NP_siCTLA-4_). The NPs effectively protected the siRNA from degradation and efficiently delivered it to T cells, consequently downregulating the expression of CTLA-4 on the T cell surface. Systemic delivery of NP_siCTLA-4_ increased the number of CD4^+^ and CD8^+^ T cells and decreased the population of CD4^+^FOXP3^+^ regulatory T cells. Moreover, NP_siCTLA-4_ effectively inhibited tumor growth and prolonged the survival of mice with melanoma tumors 84.

Programmed cell death protein -1 (PD-1) and its ligand (PD-L1) are also involved in T-cell activation through the CD28 pathway. Upon activation by PD-L1 overexpressed on DCs, PD-1 on T cells suppresses T-cell activation by inhibiting CD28 signaling 85. Hobo et al. constructed siRNA-LNPs that mediated knockdown of PD-L1 on DCs. The engineered DCs boosted the expansion of both CD4^+^ T helper cell responses and CD8^+^ effector-memory T cells 86. Hassannia et al. reported siRNA‐loaded chitosan-dextran sulfate NPs that silenced the expression of PD‐L1 on DCs and PD‐1 on T cells. The synthesized NPs demonstrated efficient cellular uptake and target gene silencing. Presentation of TAs by PD-L1-negative DCs to PD-1-silenced T cells induced potent T-cell responses 87.

### Cytokines

The signal 3 cytokines, such as interleukin (IL) 12, IL‐1, and interferon (IFN) α/β, also play a crucial role in T-cell activation 88-91. Due to their poor stability, cytokines must be administrated in large doses which often causes severe toxicities 8, 91. To address this problem, Wang et al. conjugated IFNα to a class of biocompatible, biodegradable, and thermosensitive biopolymers called elastin-like polypeptides (ELPs). The obtained IFNα-ELP_diblock_ conjugates self-assembled into nanomicelles with IFNα tightly wrapped in the corona. Due to steric hindrance, IFNα was protected from degradation by proteases. In addition, the blood circulation of IFNα in the micelles was prolonged compared to free IFNα because of its improved proteolytic stability and increased size, effectively reduced renal clearance 92. Tang et al. developed a TCR-signaling-responsive NP that controlled cytokine delivery in response to T-cell activation. The authors used human interleukin-15 super-agonist (IL-15SA) as a model drug cargo. The safe dose of IL-15SA was increased 8 times compared to free cytokine through the regulated drug release, increasing the therapeutic window for adjuvant cytokine therapy 93.

Activation of the stimulator of interferon genes (STING) pathway within tumor-resident DCs can induce the production of type I IFNs and adaptive immune responses against tumors 94, 95. Current STING agonists, such as cyclic dimeric guanosine monophosphate (cdGMP), amidobenzimidazole, and 5,6-dimethylxanthenone-4-acetic acid (DMXAA), are limited by inefficient TDLN-targeting and uncontrollable systemic inflammation. Systemic administration of these immunotherapeutic agents can influence the function of immune cells at nontarget sites, breaking immune homeostasis and causing undesirable adverse events. As mentioned above, nanotechnology could reduce off-target distribution and so decrease systemic side effects. Hanson et al. encapsulated cdGMP within PEGylated LNPs (NP-cdGMP), which effectively improved the accumulation of cdGMP in TDLNs. Compared with free cdGMP, NP-cdGMP demonstrated increased CD8^+^ T-cell responses and enhanced therapeutic antitumor immunity 96.

In summary, nanotechnology greatly improves the efficacy and safety of cytokine therapies by protecting these vulnerable agents, reducing their non-specific biodistribution, and endowing them with specific LN-targeting ability.

## CTL trafficking and infiltration into the tumor site

Once activated, effector T cells traffic through the bloodstream and infiltrate into the tumor site. This process is usually restricted by two types of obstacles. First, “signal barriers”, such as over-expression of immunosuppressive signals, or lack of recruiting cytokines, restrict the homing of CD8^+^ T cells to the tumor 60. In addition, unlike hematological malignancies, solid tumors often build up “physical barriers”, such as deficient tumor vasculature, abundant cancer-associated fibroblasts (CAFs), and dense extracellular matrix (ECM) that restrain infiltrating CTLs 97, 98. Given these obstacles, strategies aimed at eliminating signal barriers and physical barriers have been developed and are often used as adjuvant therapies to enhance checkpoint blockade immunotherapy.

### Eliminating signal barriers

Chemokines are one of the major factors governing the homing of immune cells to tumors. For example, intratumoral infiltration of T cells can be inhibited by C-X-C motif chemokine ligand 12 (CXCL12) 99, 100. Nanotechnology-based gene therapy has been widely used to modulate the expression of chemokines with the distinct advantages mentioned above. For example, Goodwin et al. developed a lipid calcium phosphate (LCP) NP to deliver plasmid DNA (pDNA) encoding an engineered CXCL12 protein trap (pCXCL12-trap). Liver-specific delivery and transient expression of pCXCL12-trap via the LCP nonviral vector directed the liver to resist infiltrating CXCR4^+^ metastatic cells. This strategy further inhibited the establishment of an iTME, allowing for enhanced cancer-specific CD8^+^ T-cell killing 101. IL-10 is also considered an immunosuppressive cytokine due to its association with suppressive and regulatory cells, including tolerogenic DCs, regulatory CD4^+^T cells, M2 macrophages, and myeloid-derived suppressor cells (MDSCs). Huang's group utilized liposome-protamine-DNA (LPD) NPs to encapsulate plasmids encoding small trap proteins that target IL-10 and CXCL12. The IL-10 trap in combination with the CXCL12 trap, significantly reduced the establishment of an iTME 102, 103.

### Eliminating physical barriers

The abnormal tumor vasculature is a critical barrier to T-cell infiltration because it provides inadequate blood perfusion and exhibits deregulated expression of the adhesion molecules required for T-cell extravasation. Therefore, treatment to normalize tumor vessels enhances CTL infiltration 104. It has been reported that nitric oxide (NO) regulates angiogenesis and maintains vascular homeostasis 105. However, most NO-delivery agents are limited by their short half-life, uncontrollable NO release, and poor tumor targeting. Sung et al. reported biodegradable lipid-PLGA NPs (denoted as NanoNO) encapsulating the NO donor dinitrosyl iron complex (DNIC). NanoNO avoided recognition by macrophages and interaction with serum proteins and demonstrated controlled release of NO. Moreover, NanoNO showed increased tumor accumulation compared with free DNIC due to the EPR effect. Thus, encapsulation of DNIC into NanoNO improved its stability, half-life, and tumor accumulation. In addition, after penetrating the TME and entering cancer cells, the pH-sensitive NanoNO specifically release NO in the acidic endosomes/lysosomes. The released NO reprogrammed the gene expression profile of endothelial cells, shifting them from a pro-angiogenic phenotype to a vascular-stabilizing signature. Therefore, treatment of mice bearing HCC tumors with NanoNO facilitated significant CD4^+^ and CD8^+^ T-cell infiltration 106.

CAFs are a subset of fibroblasts that are perpetually active in tumors and are one of the most crucial TME components 107. The dense ECM produced by CAFs creates high interstitial fluid pressure, which is a physical barrier to T-cell infiltration 108. CAFs also mediate T-cell exclusion, which prevents cancer cells from physically contacting CTLs. Therefore, killing CAFs could enhance the efficiency of immunotherapy 109. Fibroblast-activation protein (FAP), which is overexpressed on the surface of CAFs, has been proposed as a universal tumor-targeting antigen. Zhen et al. reported a CAF-targeted nanoparticle-based photo-immunotherapy (nano-PIT). Ferritin, a compact nanoparticle protein cage, was exploited as a photosensitizer carrier and conjugated with a FAP-specific single-chain variable fragment (scFv) on its surface. These nanoconjugates selectively homed to CAFs in tumors. Upon laser irradiation, activated nano-PIT efficiently eliminated the CAFs. In response, the ECM surrounding the tumor was destroyed, leading to significantly enhanced CD8^+^ T-cell infiltration 110. Many studies have demonstrated the benefit of CAF depletion for enhancing T-cell infiltration. Nonetheless, preclinical and clinical trials have also reported that depletion of CAFs could accelerate tumor progression and metastasis. These studies illustrate the need for a critical and comprehensive evaluation of CAF depletion 111, 112.

**Figure 4:**
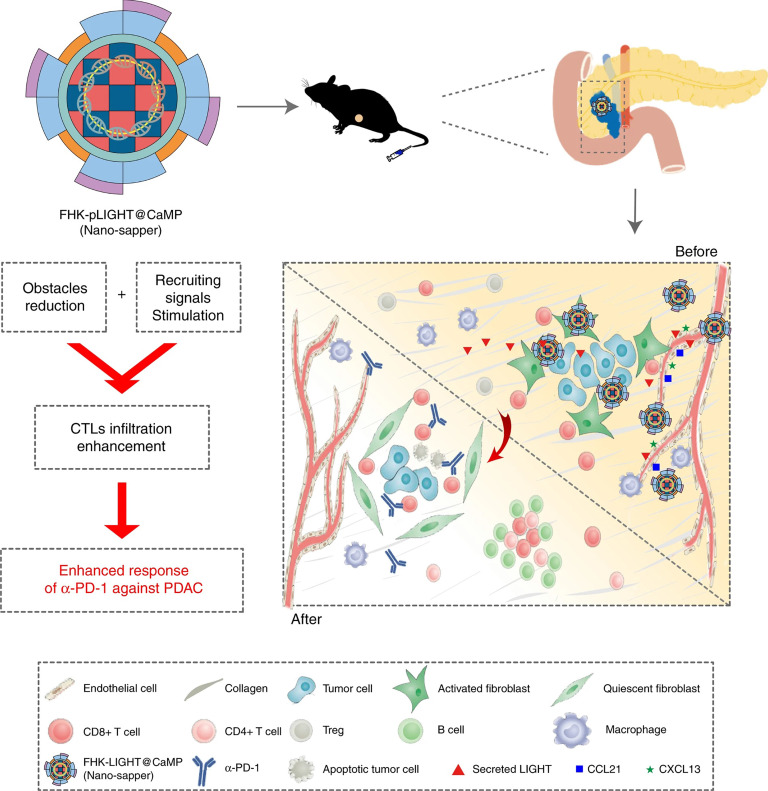
The expected effects of Nano-sapper synergized with immune-checkpoint inhibitor. Nano-sapper specifically modulate the TME of PDAC, which involves the reduced physical barrier (attenuated stroma and normalized vessels) and the release of chemoattractants recruiting lymphocytes (CCL21 and CXCL13). Once the TME has been reprogrammed by Nano-sapper, a variety of adaptive immune cells migrate to the PDAC and enhance the anti-tumor effects of α-PD-1. Adapted with permission from 113, Copyright 2020 Springer Nature.

To optimally enhance the tumor infiltration of CTLs, strategies that address both signal barriers and physical barriers are needed. Huang et al. proposed an infiltration enhancement strategy that synergistically breaks the physical obstacles and increases recruiting signals in the iTME. This synergistic effect was achieved by combining phosphate-modified α-mangostin (MP) and a plasmid encoding a pleiotropic inflammatory cytokine (pLIGHT). MP is a natural small molecule that reduces fibrogenesis and decreases collagen deposition, while pLIGHT normalizes defective vessels and facilitates T-cell recruitment. MP and pLIGHT were co-loaded into an ECM glycoprotein (tenascin C) targeted peptide (FHK)-decorated calcium phosphate liposome (denoted as FHK-pLIGHT@CaMP), as shown in Figure 4. Decoration of the liposome with the FHK peptide enhanced the tumor retention of the loaded components. Then, MP reversed the CAFs activation, decreased collagen deposition, and relieved compressed vessels. The secreted LIGHT recovered vessel functions and stimulated the expression of lymphocyte-recruiting chemo-attractants. Accordingly, MP and LIGHT synergistically improved CTL infiltration and supported local generation of a tumor-specific immune response 113.

In conclusion, NPs with good stability, long circulation, and efficient tumor targeting could greatly assist the tumor infiltration of T cells. These strategies could further improve the outcomes of immunotherapies such as adoptive T-cell therapy and anti-PD-1/anti-PD-L1 therapy, which constantly suffer from a low response rate due to the T-cell infiltration issue 98, 114.

## CTL recognition and killing of tumor cells

Finally, depending on TCR and MHC interactions, effector T cells specifically recognize tumor cells and eliminate them by releasing granzymes and perforins 5. However, once T cells finally infiltrate into the iTME, they are still prevented from effectively recognizing and killing tumor cells, due to binding of PD-L1 (expressed on the tumor cell surface) with PD-1 (expressed on the T cell surface). Additionally, the iTME may protect tumor cells against potent CTL recognition and killing, preventing CTLs from eliminating tumor. Strategies aimed at enhancing CTL recognition and killing of tumor cells have been assessed in clinical trials. These strategies include immune checkpoint blockade (ICB) of PD-1/PD-L1, chimeric antigen receptor (CAR) T-cell therapy (which endows T cells with antibody recognition specificity), and rebuilding the iTME.

### Disrupting the PD-1/PD-L1 pathway

**Figure 5:**
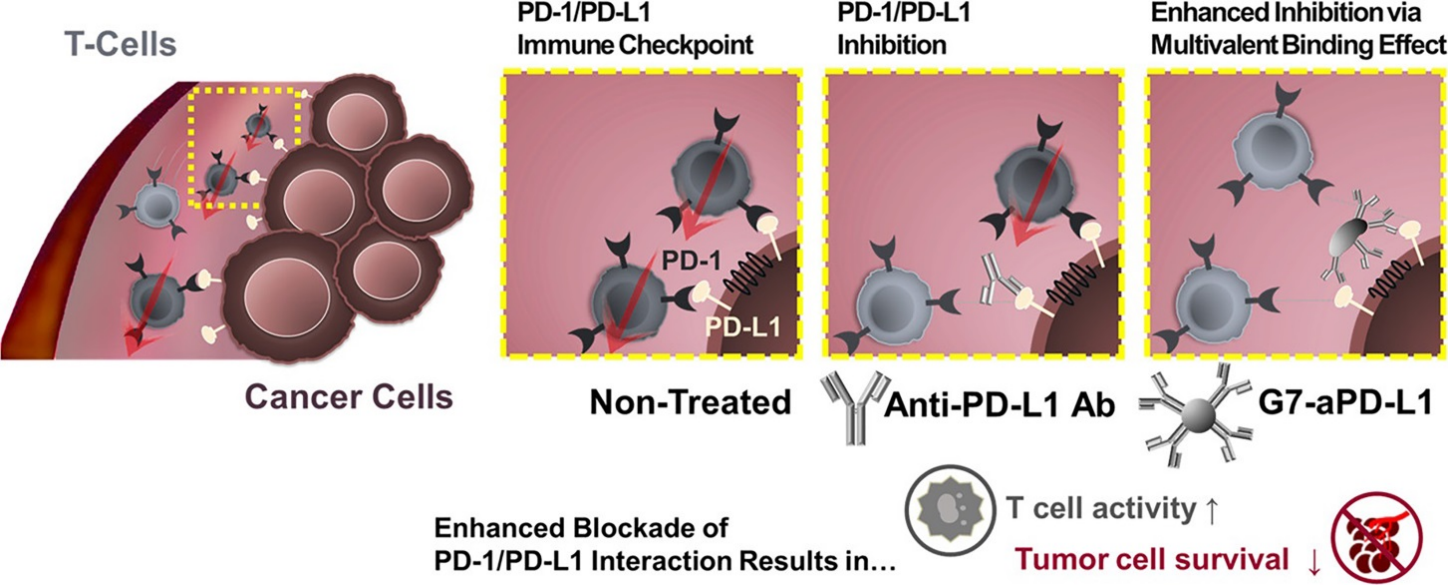
A schematic diagram illustrating the hypothesis that the dendrimer-mediated multivalent interaction would substantially increase the antagonist effect of ICIs as a result of increased binding kinetics. Adapted with permission from 117, Copyright 2020 American Chemical Society.

Except for attenuating T cell activation, the interaction between PD-1 on T cells with PD-L1 on tumor cells induces effector T cell apoptosis, anergy, and functional exhaustion 85. PD-L1 on tumor cells is widely used as a biomarker for ICB 115. Despite exciting clinical results, the clinically used PD-1/PD-L1-monoclonal antibody still faces considerable challenges, such as low response rate 116, low binding strength, and unmanageable side effects. Unfortunately, clinical translation of small molecular inhibitors is also restricted by their rapid clearance and poor tumor accumulation. For these reasons, nanotechnology has been applied to improve the therapeutic efficacy of PD-1/PD-L1 inhibitors. Bu et al. conjugated generation 7 (G7) poly(amidoamine) (PAMAM) dendrimers with PD-L1 targeting molecules to generate G7-aPD-L1 (Figure 5). The dendrimer NPs formed multiple binding pairs with PD-L1 proteins, creating significantly stronger interactions with the target receptors than free aPD-L1. This enhancement in binding kinetic increased the effects of the PD-L1 antagonist 117.

Systemic inhibition of PD-L1 risks breaking peripheral tolerance, causing autoimmune diseases and safety issues 18, 118, 119. Thus, intelligent nanocarriers that can distinguish tumors from normal tissues have been developed to achieve tumor-specific drug delivery. Acidic microenviroment (pHe) and ROS overexpression, especially hydrogen peroxide (H_2_O_2_), are two typical characteristics of solid tumors. Zhang et al. constructed a dual-locking nanoparticle (DLNP) with clustered regularly interspaced short palindromic repeat (CRISPR)/CRISPR-associated (Cas) enzyme Cas13a 120. The DLNP had a core-shell structure with the CRISPR/Cas13a system pDNA targeting PD-L1 encapsulated inside the core, and a pHe/H_2_O_2_ dual-responsive polymer layer as the shell. In blood circulation and normal tissues, the polymer layer endowed the DLNP with a negatively charged and PEGylated surface, contributing to its stability. Upon reaching the TME, the polymer layer was degraded into a cationic polymer, facilitating cellular internalization and PD-L1 inhibition. This approach reduced the side effects of the immunotherapy by avoiding undesired PD-L1 inhibition in normal tissues 121.

Apart from PD-1/PD-L1, inhibitory molecules that restrain the activity of CTLs, include lymphocyte-activation gene-3 (Lag-3), T cell immunoglobulin-3 (TIM-3), T-cell immunoreceptor with Ig and ITIM domains (TIGIT), and V-domain Ig suppressor of T-cell activation (VISTA) 122-125. Most of these are under development in clinical and preclinical studies and show synergistic effects in combination with blockade of the PD-1/PD-L1 pathway. However, such studies are rarely reported in combination with nanotechnology 33.

### CAR-T therapy

Adoptive cell therapy (ACT) is a personalized therapy in which a patient's immune cells are expanded *in vitro* to large numbers and then reinfused to eradicate tumors 126. Genetic modification of T cells with CARs is the most promising ACT strategy. In this strategy, a patient's T cells are transfected with a construct encoding an antibody against a tumor surface antigen (typically CD19), thereby endowing them with the specificity of antibody-like recognition 127. However, the generation and storage of large numbers of CAR T cells is too complicated for industrial production and clinical application. Nanocarriers with simple preparation, good stability, and T cell-targeting ability could solve these problems. Hence, Smith et al. demonstrated that DNA-carrying NPs could efficiently introduce leukemia-targeted CAR genes into T-cell nuclei. The authors chose poly (β-amino ester) as the core material for the T cell-targeted nanocarriers. NPs carrying genes of CD19-specific CARs selectively and rapidly edited T-cell specificity *in vivo*. In addition, these polymer NPs were easy to manufacture in a stable form, which simplified storage and reduced costs, and demonstrated transfection efficacies comparable to that of the conventional adoptive transfer of laboratory-manufactured CAR T cells 128.

### Modulation of iTME

The TME contains a network of immunosuppressive factors that pose a formidable barrier to CTLs 98. Reprogramming of immunosuppressive factors in the iTME is essential. MDSCs and M2 macrophages are the main immunosuppressive immune cells promoting tumor growth. Inhibition of phosphoinositide-3-kinases (PI3Ks), which are pivotal for the function of myeloid cells, can effectively reshape the iTME. Zhang et al. designed aminoethylanisamide (AEAA)-modified polymeric NPs to encapsulate IPI-549, an oral PI3K-γ inhibitor in clinical development. The engineered IPI-549 NPs exhibited efficient cellular uptake, a long half-life, and strong tumor accumulation. The IPI-549 NPs induced the reduction of immunosuppressive cells, such as regulatory B cells and MDSCs, and changed the transcription factor and cytokines, such as IL-10 and IFN-γ, thereby remodeling the iTME 129. Tumors often stimulate tumor-associated macrophages (TAMs) to display an immunosuppressive M2 phenotype, which supports tumor growth through the production of cytokines such as IL-10, instead of the anti-tumorigenic M1-like phenotype. A great amount of effort has been dedicated to either depleting M2-like TAMs or converting their phenotype into tumoricidal M1-like TAMs. Rodell et al. screened out a potent driver of the M1 phenotype, R848 (an agonist of TLR7/8). The authors constructed β-cyclodextrin nanoparticles (CDNPs) to deliver R848. CDNP-R848 displayed high TAM affinity and high drug-loading capacity due to the covalent crosslinking of the CD. Administration of CDNP-R848 in mice altered the functional orientation of TAMs towards an M1 phenotype, leading to controlled tumor growth and protection against tumor rechallenge 130.

In addition to their immunostimulatory functions, TLR7/8 agonists (TLR7/8a) can also modulate the iTME by transforming MDSCs into APCs such as DCs and macrophages, and polarizing TAMs from M2 to M1. Kim et al. encapsulated TLR7/8a in the core of a nanoemulsion (NE) to improve its pharmacokinetic properties and reduce systemic toxicity. Administration of NE (TLR7/8a) induced recruitment and activation of innate immune cells, infiltration of lymphocytes, and polarization of M2-like TAMs, thus reprogramming the iTME 131. Liang et al. used triblock copolymer NPs to co-deliver the STING agonist DMXAA and SN38. The prepared NPs (named PS3D1@DMXAA) enabled efficient intracellular delivery of DMXAA. PS3D1@DMXAA enhanced antigen cross-presentation and induced conversion of the iTME to an immunogenic TME through the synergistic interaction between SN38 and STING activation 132.

**Figure 6:**
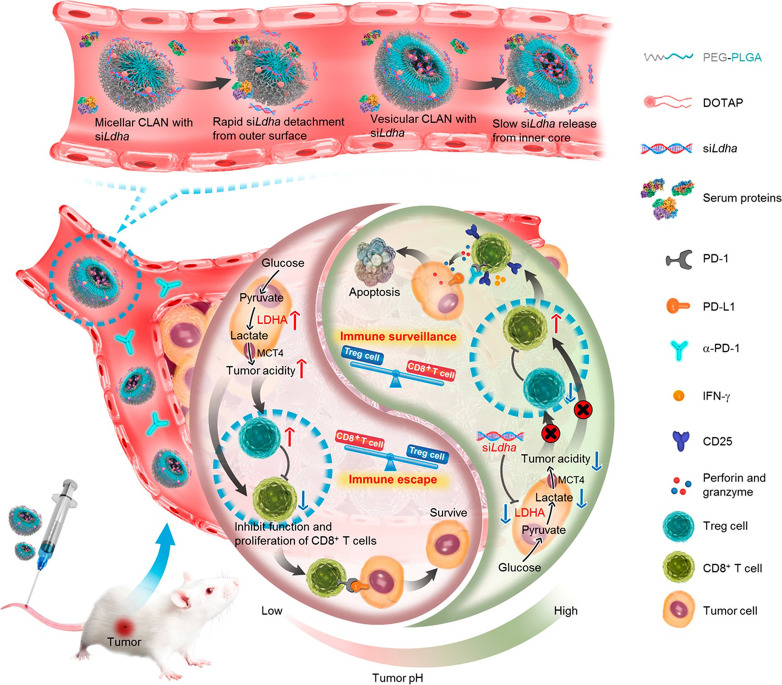
Nanotechnology-mediated reversion of tumor immunosuppressive microenvironment through tumor acidity modulation. The pH of the tumor microenvironment modulated the activation and proliferation of infiltrating immune cells and thereby regulated the balance between the immune surveillance and escape (also known as immune response and tolerance). Tumor cells overexpressing LDHA converted glucose into lactate, which blunted tumor surveillance by T cells. Nanotechnology-mediated knockdown of LDHA therapeutically reversed the tumor acidic immunosuppressive microenvironment, which decreased the number of immunosuppressive cells, increased infiltration of CD8^+^ T cells, and restored their anti-tumor functions. Tumor acidity neutralization prior to α-PD-1 checkpoint blockade therapy improved the anti-tumor response and produced synergistic effects. Adapted with permission from 134, Copyright 2019 American Chemical Society.

Tumor acidity plays an immunosuppressive role in impeding effective antitumor T-cell immune responses. Specifically, CD8^+^ T cells tend to become anergic when exposed to a low pH environment. In addition, excessive lactate, the product of aerobic glycolysis, enhances the function of immunosuppressive cells and thereby blunts antitumor immune responses 133. Hence, antagonizing tumor acidity may reverse the detrimental effects of lactate and recover the functions of antitumor T cells. As shown in Figure 6, Zhang et al. utilized vesicular cationic lipid-assisted nanoparticles (CLAN) to mediate the knockdown of lactate dehydrogenase A (LDHA) in tumor cells. CLAN prevented premature release of encapsulated siRNA during blood circulation and improved the tumor accumulation of siRNA. CLAN-mediated gene silencing efficiently downregulated LDHA expression, decreased lactate secretion, and raised the tumor pH. In immunocompetent syngeneic melanoma and breast tumor models, neutralization of tumor acidity increased infiltration of CD8^+^ T and NK cells, decreased the number of immunosuppressive T cells, and thus significantly inhibited tumor growth 134.

In summary, with advantages including long circulation, strong tumor accumulation, efficient cellular uptake, and intelligent drug release, advanced nanocarriers can be a great strategy to reduce immunosuppressive cytokines/cells, remodel the iTME, and ultimately restore T-cell function.

## Combination nano-immunotherapy

Even though the above nano-immunotherapies have demonstrated efficiency in preclinical studies, only a minority of patients can benefit from them. This failure is mainly because most of these therapies only focus on a single step of the cancer-immunity cycle, which is insufficient when the cycle is affected by multiple factors. Combination immunotherapy is one way forward 135-137. However, combination immunotherapy is challenged by inefficient co-delivery and dangerous autoimmune reactions 114. In addition, since immunotherapy agents have different properties in terms of pharmacokinetics, biodistribution, and mechanism of action 6, it may not be easy to optimize the synergistic effect. NPs have emerged as a promising carrier for the co-delivery of multiple agents. Advanced nanocarriers can co-deliver multiple drugs with different physicochemical properties through physical adsorption, hydrogen bonding or chemical bonding 138-140. Beyond this advantage, LN/tumor-targeted NPs can limit the exposure of drugs to normal tissues, thereby improving therapeutic efficacy while reducing systemic toxicity 141, 142.

### Combination therapy with enhanced efficacy

NPs can deliver multiple drugs with different properties, contributing to effective synergetic therapy. Indoleamine 2,3-dioxygenase (IDO) inhibitors, like NLG-8189, have poor clinical efficacy due to their unsatisfactory pharmacokinetic profiles and tumor accumulation. Since, single use of IDO inhibitors has limited efficacy, combination with other therapies is necessary. Thus, NPs have been used to improve the bioactivity of IDO inhibitors and achieve effective co-delivery. Shen et al. prepared a bifunctional liposome to co-deliver intravenous oxaliplatin (Oxa [IV]) and NLG-919. To improve their encapsulation efficiencies, Oxa (IV) and NLG-919 were converted into prodrugs via conjugation with the phospholipid DSPE and dodecanoyl chloride, respectively. The amphiphilic Oxa (IV) prodrug and the hydrophobic NLG-919 prodrug self-assembled into aNLG/Oxa (IV)-Lip, achieving effective co-loading of NLG-919 and Oxa (IV). The obtained NPs released cytotoxic Oxa (IV) inside the reductive cytosol, triggering ICD of cancer cells. Additionally, the NPs efficiently retarded the degradation of tryptophan and reduced immunosuppressive kynurenine via NLG-919-mediated inhibition of IDO1. Furthermore, the NPs exhibited a long blood circulation time, thereby enabling efficient passive tumor targeting. As a result, the NPs presented synergistic antitumor efficacy, contributing to enhanced intratumoral infiltration of CD8^+^ T cells, secretion of cytotoxic cytokines, and downregulation of immunosuppressive regulatory T cells 143.

### Combination therapy with reduced toxicity

NPs can reduce the distribution of immunotherapeutic drugs in normal tissues and allow them to specifically accumulate in target tissues, which is of great significance for reducing systemic toxicity. Chiang et al. reported a nanomedicine (IO@FuDex) composed of superparamagnetic iron oxide nanoparticles (IO), fucoidan (Fu), and aldehyde-functionalized dextran (Dex). IO@FuDex functionalized with anti-PD-L1 and the T-cell activators anti-CD3 and anti-CD28 (IO@FuDex^3^) achieved simultaneous PD-1/PD-L1 checkpoint inhibition and T-cell proliferation. Moreover, IO@FuDex^3^ could be localized to the desired site of action by application of an external magnetic field, which significantly reduced systemic accumulation. The combination of IO@FuDex^3^ and magnetic navigation decreased the occurrence of adverse events 144.

## Conclusions, challenges, and perspectives

The ultimate goal of anticancer immunotherapy is to eliminate tumors and enable patients to maintain tumor immunity for a long time. At present, due to low efficacy and high risk of immune-mediated toxicities, anticancer immunotherapy is far from achieving this goal. The combination of nanotechnology and immunotherapy, named nano-immunotherapy, has brought opportunities to solve many of these problems. However, the complexity and variability of the host immune system pose great challenges to efficient nano-immunotherapy. Therefore, “which” immune step is targeted, “why” it needs to be further enhanced, and “what” nanotechnology can do for immunotherapy must be fully considered when developing nano-immunotherapies.

To answer these questions, we summarizd the current developments in nano-immunotherapy based on the concept of the cancer-immunity cycle. We divided cellular immunity into two stages and four parts. Immunotherapies in the preparatory stage cover the field of TA release and presentation (steps 1 and 2) and T-cell priming and activation (step 3), and include ICD induction, CD47 blockade, aAPCs, CTLA-4 blockade, and administration of inflammatory stimulating cytokines. These immunotherapies usually use sensitive antigens or proteins that are easily degraded or inactivated by enzymes in complex physiological environments. Nanotechnologies provide strong protection for these agents, increasing their half-lives, minimizing systemic toxicity, and promoting their delivery to APCs. Moreover, LNs are the “base camp” in the preparatory stage, where the anticancer immune response is initiated. Due to their suitable size and particular structure, NPs have the promising potential of achieving adequate drainage and retention in LNs, thus enhancing the immune response in the preparatory stage.

Nano-immunotherapies in the effector stage involve CTL trafficking and infiltration into the tumor site (steps 4 and 5) and CTL recognition and killing of tumor cells (steps 6 and 7), and include elimination of TME barriers, blockade of the PD-1/PD-L1 pathway, removal of iTME immunosuppression, and CAR-T therapy. NPs have prolonged systemic circulation and tumor targeting effect capabilities. In addition, specific NPs can control drug release in response to typical characteristics of the TME, such as hypoxia and low pH. All these advantages of nanotechnology could help to enhance the therapeutic effects of immunotherapies and decrease their side effects.

It has been reported that combination immunotherapy is more effective than monotherapy in cancer treatment. Combinations of tumor vaccines, immune checkpoint inhibitors, ACTs, and TME regulatory treatments have been well studied 57, 145, 146. Despite the increased therapeutic benefits, the risks of autoimmune responses, toxicities, and adverse events are also greater for combination therapy than monotherapy. In addition, effective co-delivery of immunotherapy agents with different properties and mechanisms is also challenging. NPs that co-encapsulate multiple drug molecules are promising platforms to overcome the barriers of combination therapy. However, with synthetic carrier materials and complex preparation methods, combination nano-immunotherapy faces still greater challenges in clinical translation.

As new opportunities always come with new challenges, nanotechnology brings additional challenges to immunotherapy. The EPR effect has been regarded as a golden rule of anticancer nanomedicine and has been well-studied in animal models with xenografted tumors. However, xenograft tumors are significantly different from human cancers. Due to the complexity and heterogeneity of human tumors, as well as ethical issues with conducting experiments in patients, the EPR effect in human tumors has not yet been fully investigated and its clinical relevance remains controversial 17, 142. Thus, whether the EPR effect in humans can effectively facilitate nano-immunotherapy needs to be reconsidered. In addition, nanotechnology increases the risk of overdriving the immune system, and nanomaterials also suffer from safety issues. The potential immunogenicity of nanomaterials is a double-edged sword for immunotherapy. On the one hand, some nanomaterials themselves can induce inflammatory responses and promote or enhance immune responses against cancer. On the other hand, if a nano-immunologic agent is recognized as foreign substance and opsonized by plasma proteins, the complement pathway is activated, resulting in rapid phagocytosis and clearance of the drug by the liver and spleen 147. Complete activation of the immune response may lead to serious complications, including allergic reactions, hemolysis, thrombogenesis, and even disseminated intravascular coagulation 16, 98. What is more, there are critical concerns about the quality of nano-immunologic agents, the fate of nanomedicines in the body, and interactions with immune organs and the TME, which need to be comprehensively studied and tested.

## Abbreviations

LNs: lymph nodes
TME: tumor microenvironment
iTME: immunosuppressive tumor microenvironment
NPs: nanoparticles
EPR effect: the enhanced permeation and retention effect
APCs: antigen presenting cells
TAs: tumor antigens
CTL: cytotoxic T lymphocyte
DCs: dendritic cells
MHC: major histocompatibility complex
ETAs: endogenous tumor antigens
PDT: photodynamic therapy
PTT: photothermal therapy
ER: endoplasmic reticulum
MSNs: mesoporous silica nanoparticles
Fe_3_O_4_: iron oxide
ROS: reactive oxygen species
Ce6: chlorin e6
ICD: immunogenic cell death
DAMPs: damage associated molecular patterns
CRT: calreticulin
HMGB1: high mobility group box 1
ATP: adenosine triphosphate
Q: quercetin
A: alantolactone
PM: plasma membrane
PCPK: PpIX-C6-PEG8-KKKKKKSKTKC-OMe
PFTase: protein farnesyltransferase
TDLNs: tumor draining lymph nodes
Si-OH: hydrophilic silanol groups
OVA: ovalbumin
PRRs: pattern recognition receptors
TLR: Toll-like receptor
InAc: inulin acetate
PLGA: poly(lactic-co-glycolic acid)
LNPs: lipid nanoparticles
SIRPα: signal regulatory proteinα
CD47nb: nanobody antagonist of CD47
pMHC: peptide-MHC
aAPCs: artificial APCs
TCR: T cell receptor
CTLA-4: cytotoxic T-lymphocyte associated antigen 4
siCTLA-4: CTLA-4 small interfering RNA
PEG-PLA: poly(ethylene glycol)-polylactide
PD-1: programmed cell death-1
PD-L1: programmed death-ligand 1
IL: interleukin
IFN: interferon
ELPs: elastin-like polypeptides
IL-15SA: IL-15 super-agonist
STING: Stimulator of Interferon Genes
cdGMP: cyclic di-GMP
DMXAA: 5,6-dimethylxanthenone-4-acetic acid
CAFs: carcinoma-associated fibroblasts
ECM: extracellular matrix
CXCL12: C-X-C motif chemokine ligand 12
LCP: lipid calcium phosphate
pDNA: plasmid DNA
MDSCs: myeloid-derived suppressor cells
LPD: liposome-protamine-DNA
NO: nitric oxide
DNIC: dinitrosyl iron complex
FAP: fibroblast-activation protein
nano-PIT: nanoparticle-based photo-immunotherapy
scFv: single-chain variable fragment
IL-10: Interleukin-10
MP: phosphate of α-mangostin
pLIGHT: plasmid encoding pleiotropic inflammatory cytokine
ICB: immune checkpoint blockade
CAR: chimeric antigen receptor
pHe: acidic microenvironment
H_2_O_2_: hydrogen peroxide
CRISPR: clustered regularly interspaced short palindromic repeat
Lag-3: lymphocyte activation gene-3
TIM-3: T cell immunoglobulin-3
TIGIT: T cell immunoreceptor with Ig and ITIM domains
VISTA: V-domain Ig suppressor of T cell activation
ACT: adoptive cell therapy
PI3Ks: phosphoinositide-3-kinases
AEAA: aminoethyl anisamide
TAMs: tumor-associated macrophages
CDNPs: cyclodextrin nanoparticles
CLAN: cationic lipid-assisted nanoparticles
LDHA: lactate dehydrogenase A
TLR7/8a: TLR7/8 agonists
IDO: indoleamine 2,3-dioxygenase
